# Supplementary material for: Context-aware deconvolution of cell–cell communication with Tensor-cell2cell
Source: Nat Commun. 2022 Jun 27;13:3665. doi: 10.1038/s41467-022-31369-2 (PMC9237099; doi:10.1038/s41467-022-31369-2)
Supplement: Supplementary file 3 — Reporting Summary [file 41467_2022_31369_MOESM3_ESM.pdf]

nature portfolio

Corresponding author(s): Nathan E. Lewis

Last updated by author(s): May 19, 2022

Reporting Summary

Nature Portfolio wishes to improve the reproducibility of the work that we publish. This form provides structure for consistency and transparency in reporting. For further information on Nature Portfolio policies, see our Editorial Policies and the Editorial Policy Checklist.

Statistics

For all statistical analyses, confirm that the following items are present in the figure legend, table legend, main text, or Methods section.

n/a	Confirmed	
☐	☒	The exact sample size (*n*) for each experimental group/condition, given as a discrete number and unit of measurement
☐	☒	A statement on whether measurements were taken from distinct samples or whether the same sample was measured repeatedly
☐	☒	The statistical test(s) used AND whether they are one- or two-sided<br>*Only common tests should be described solely by name; describe more complex techniques in the Methods section.*
☐	☒	A description of all covariates tested
☐	☒	A description of any assumptions or corrections, such as tests of normality and adjustment for multiple comparisons
☐	☒	A full description of the statistical parameters including central tendency (e.g. means) or other basic estimates (e.g. regression coefficient) AND variation (e.g. standard deviation) or associated estimates of uncertainty (e.g. confidence intervals)
☐	☒	For null hypothesis testing, the test statistic (e.g. F, t, r) with confidence intervals, effect sizes, degrees of freedom and P value noted<br>*Give P values as exact values whenever suitable.*
☒	☐	For Bayesian analysis, information on the choice of priors and Markov chain Monte Carlo settings
☒	☐	For hierarchical and complex designs, identification of the appropriate level for tests and full reporting of outcomes
☐	☒	Estimates of effect sizes (e.g. Cohen's *d*, Pearson's *r*), indicating how they were calculated

Our web collection on statistics for biologists contains articles on many of the points above.

Software and code

Policy information about availability of computer code

Data collection	No software was used to collect data.
Data analysis	All codes for the analyses are available in a reproducible Code Ocean capsule (https://doi.org/10.24433/CO.0051950.v2), including exact versions of tools and software). Tensor-cell2cell was implemented as a part of the cell2cell suite for inferring cell-cell interactions (https://github.com/earmingol/cell2cell), which is available for Python. While the analyses for measuring computational efficiency are available in a github for running them locally (https://github.com/LewisLabUCSD/CCC-Benchmark).<br><br>Versions of tools and software are:<br>Python 3.8.1 and packages:<br>anndata==0.7.4<br>cell2cell==0.5.9<br>gseapy==0.10.8<br>h5py==2.9.0<br>kneed==0.7.0<br>matplotlib==3.2.0<br>numba==0.51.2<br>numpy==1.19.2<br>openpyxl==3.0.3<br>python-igraph==0.7.1.post6<br>scanorama==1.7.1<br>scanpy==1.6.0<br>scikit-learn==0.23.2

```
  scipy==1.5.2
  seaborn==0.11.0
  statannot==0.2.3
  statsmodels==0.12.1
  tensorly==0.5.1

R 4.1.2 and libraries from github repos:
  igraph/rigraph@master
  keepsimpler/StabEco
  saezlab/liana
  sqjin/CellChat
```

For manuscripts utilizing custom algorithms or software that are central to the research but not yet described in published literature, software must be made available to editors and reviewers. We strongly encourage code deposition in a community repository (e.g. GitHub). See the Nature Portfolio guidelines for submitting code & software for further information.

Data

Policy information about availability of data

All manuscripts must include a data availability statement. This statement should provide the following information, where applicable:

- Accession codes, unique identifiers, or web links for publicly available datasets
- A description of any restrictions on data availability
- For clinical datasets or third party data, please ensure that the statement adheres to our policy

All input data used for the analyses in this work and the result-generated data are available online in a Code Ocean capsule (https://doi.org/10.24433/CO.0051950.v2). In particular, we used a single-cell atlas of COVID-19 in humans, previously deposited in the NCBI's Gene Expression Omnibus database under accession code GSE158055 [https://www.ncbi.nlm.nih.gov/geo/query/acc.cgi?acc=GSE158055], a COVID-19 dataset of single-cell transcriptomes for BALF samples, previously deposited in the NCBI's Gene Expression Omnibus database under accession code GSE145926 [https://www.ncbi.nlm.nih.gov/geo/query/acc.cgi?acc=GSE145926], and a single-nucleus ASD dataset previously deposited in the NCBI's BioProject database under accession code PRJNA434002 [https://www.ncbi.nlm.nih.gov/bioproject/PRJNA434002/]. The list of ligand-receptor interactions employed in our analyses corresponds to the database previously published with CellChat, and is available in a Compendium of Ligand-Receptor Pairs [https://github.com/LewisLabUCSD/Ligand-Receptor-Pairs/blob/master/Human/Human-2020-Jin-LR-pairs.csv] that we previously published. The data generated in this study for the loadings resulting from the tensor decompositions of the COVID-19 and ASD datasets are available in the Source Data file.

Field-specific reporting

Please select the one below that is the best fit for your research. If you are not sure, read the appropriate sections before making your selection.

☒ Life sciences ☐ Behavioural & social sciences ☐ Ecological, evolutionary & environmental sciences

For a reference copy of the document with all sections, see nature.com/documents/nr-reporting-summary-flat.pdf

Life sciences study design

All studies must disclose on these points even when the disclosure is negative.

Sample size	No sample-size calculation was performed. Instead, we used the number of samples included in each of the previously published datasets that we used.
Data exclusions	The only data exclusion performed was for the PBMC COVID-19 datasets, which originally includes 284 samples. For running our benchmarking, we included only 60 samples randomly selected for each COVID-19 severity, where 20 corresponded to control patients, 20 mild/moderate COVID-19 patients, and 20 severe/critical COVID-19 patients. The idea here was to keep a balance number of patients for each category.
Replication	We deposited all our analyses including data and exact versions of code and software in a Code Ocean capsule (https://doi.org/10.24433/CO.0051950.v2). With this, we make sure that our results can be replicated by running the analyses in that capsule.
Randomization	Not relevant for the study because we analyzed previously published datasets, using their corresponding samples.
Blinding	Not relevant for the study because we analyzed previously published datasets, using their previously de-identified samples.

Reporting for specific materials, systems and methods

We require information from authors about some types of materials, experimental systems and methods used in many studies. Here, indicate whether each material, system or method listed is relevant to your study. If you are not sure if a list item applies to your research, read the appropriate section before selecting a response.

Materials & experimental systems

n/a	Involved in the study
☒	☐ Antibodies
☒	☐ Eukaryotic cell lines
☒	☐ Palaeontology and archaeology
☒	☐ Animals and other organisms
☒	☐ Human research participants
☒	☐ Clinical data
☒	☐ Dual use research of concern

Methods

n/a	Involved in the study
☒	☐ ChIP-seq
☒	☐ Flow cytometry
☒	☐ MRI-based neuroimaging
